# STAT5: Understanding the Biology of KRAS^G12D^‐Driven Pancreatic Cancer

**DOI:** 10.1111/jcmm.70303

**Published:** 2024-12-19

**Authors:** Kostas A. Papavassiliou, Christos Adamopoulos, Athanasios G. Papavassiliou

**Affiliations:** ^1^ First University Department of Respiratory Medicine, ‘Sotiria’ Chest Hospital, Medical School National and Kapodistrian University of Athens Athens Greece; ^2^ Department of Biological Chemistry, Medical School National and Kapodistrian University of Athens Athens Greece; ^3^ Department of Oncological Sciences Icahn School of Medicine at Mount Sinai New York City New York USA

**Keywords:** inflammation, KRAS^G12D^, metabolic reprogramming, pancreatic cancer, STAT5

Pancreatic cancer is the 12th most frequent cancer globally, with > 500.000 cases diagnosed every year. The majority of pancreatic cancers are typically diagnosed at a late stage, with 80% not being detected until after the disease has advanced and there can be significant resistance to radiotherapy and chemotherapy. Pancreatic cancer has the lowest survival of all the 20 common cancers—over half of people with the disease die within 3 months of diagnosis. The survival of patients beyond 5 years has improved very little for some time; therefore, it is of paramount importance that we find new ways to illuminate the molecular underpinnings of this disease, how it spreads, and why it is so aggressive.

Kirsten rat sarcoma viral oncogene homologue (KRAS) mutations are among the most commonly occurring mutations in human cancers. KRAS^G12D^ is the prevalent KRAS mutation and is detected in the majority of KRAS‐mutated pancreatic tumours. A recent article by Lin et al. [1] explored the role of the transcription factor signal transducer and activator of transcription 5 (STAT5) in KRAS^G12D^‐driven pancreatic cancer. The authors revealed that, in the setting of mutant KRAS^G12D^ and chronic inflammation, STAT5 activation fosters pancreatic cancer progression through mediating metabolic reprogramming and acinar‐to‐ductal metaplasia (ADM). These data open the door to novel combinatorial therapeutic approaches directed against STAT5 and KRAS^G12D^ to improve the clinical outcome of pancreatic cancer patients. Although this study provides valuable insights into the biology of KRAS^G12D^‐induced and inflammation‐triggered pancreatic cancer, it simultaneously leaves many knowledge gaps regarding molecular mechanisms that need to be filled in order to render its findings translatable.

Understanding what drives pancreatic cancer, especially during its early steps, is of critical importance with respect to prognosis and provides an opportunity for therapeutic intervention if detected at that stage. The acquisition of somatic KRAS mutations and exposure to chronic inflammation, such as chronic pancreatitis, predispose pancreatic acinar cells to malignant transformation. Through a series of in vivo and in vitro functional experiments, Lin et al. identified STAT5 as a pivotal downstream effector of both oncogenic KRAS^G12D^ signalling and interleukin‐22 (IL‐22)‐related inflammation, binding directly to the promoter sequences of ADM‐associated genes (*hepatocyte nuclear factor 1β* (*HNF1β*) and *hepatocyte nuclear factor 4α* (*HNF4α*)) and genes engaged in energy metabolism (*HNF4α*). Accordingly, the authors reported that STAT5 enhances ductal structure formation, oxidative phosphorylation and glycolysis in pancreatic acinar cells harbouring KRAS^G12D^ mutation and being stimulated by IL‐22 (Figure 1). A previous *Cancer Discovery* article by Dey et al. [2] complements the findings by Li et al. showing that KRAS^G12D^ drives metabolic reprogramming via utilising the Janus kinase (JAK)–STAT pathway activated by upstream inflammatory signals emanating from immune cells within the tumour microenvironment (TME). However, considering the in vivo results of this study, several questions arise regarding the molecular underpinnings of STAT5 potentiation in the setting of oncogenic KRAS^G12D^ and IL‐22‐related inflammation.

**FIGURE 1:**
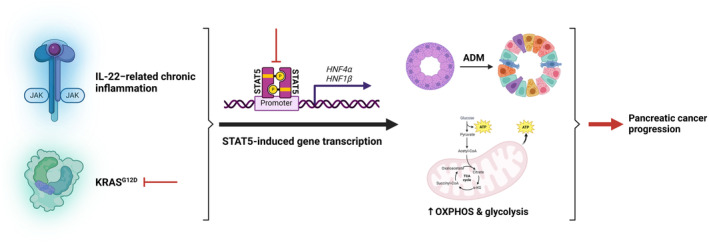
Signal transducer and activator of transcription 5 (STAT5) potentiation via oncogenic KRAS^G12D^ and interleukin‐22 (IL‐22) signalling fosters acinar‐to‐ductal metaplasia (ADM) and upregulates oxidative phosphorylation (OXPHOS) and glycolysis, resulting in pancreatic cancer progression. The red inhibitory lines represent points of therapeutic intervention through targeting with small‐molecule drugs. ATP, adenosine triphosphate; CoA, coenzyme A; HNF1β, hepatocyte nuclear factor 1β; HNF4α, hepatocyte nuclear factor 4α; JAK, Janus kinase; P, phosphorylation; TCA, tricarboxylic acid; α‐KG, alpha‐ketoglutarate. This figure was created based on the tools provided by Biorender.com (https:// biorender.com/).

First, how does KRAS^G12D^ activate STAT5? Li et al. suggest that mutant KRAS^G12D^ functions via phosphoinositide 3‐kinase (PI3K) signalling to phosphorylate and potentiate STAT5, rather than extracellular signal‐regulated kinase 1/2 (ERK1/2), nuclear factor‐κB (NF‐κB) or STAT3 signalling. Could it be that mutated KRAS causes persistent upregulation of the downstream PI3K/protein kinase B (AKT)/mechanistic target of rapamycin (mTOR) signalling cascade allowing mTOR to directly interact with and phosphorylate STAT5, as occurs in hepatocellular carcinoma? [3] Second, how does IL‐22‐mediated JAK1/2 signalling trigger KRAS^G12D^‐induced STAT5 activation? Is it possible that JAK1/2 signalling elicits STAT5 phosphorylation, resulting in its nuclear translocation and the upregulation of the expression of PI3K subunits? Third, how does oncogenic KRAS^G12D^ signalling augment the IL‐22‐dependent STAT5 activation observed in the study by Li et al.? Is there a molecular mechanism explaining the synergistic effect demonstrated between KRAS^G12D^ mutation and IL‐22 signalling? These questions need to be answered in future studies so as to advance our understanding of KRAS^G12D^‐driven pancreatic cancer. Another aspect that is not clarified by the authors of this study and needs to be addressed, concerns STAT5. STAT5 refers to two proteins, STAT5A and STAT5B, that are nearly identical in terms of amino acid sequence (94% homology), but evidence indicates that the two isoforms may function in different ways. For example, STAT5B seems to promote gemcitabine chemoresistance, cell adherence and invasiveness in pancreatic cancer cells [4]. Another study showed that STAT5B supports tumour growth, angiogenesis and metastasis in pancreatic cancer [5]. Additionally, data on different types of cancer, such as leukaemia, also highlight the functional differences between STAT5A and STAT5B [6]. Therefore, given that Li et al. do not probe the effects of the two STAT5 isoforms separately, we cannot draw conclusions on whether both proteins or only one of them contributes to KRAS^G12D^‐ and inflammation‐linked pancreatic carcinogenesis.

Notwithstanding, the study by Li et al. uncovers vital knowledge on pancreatic cancer driven by oncogenic KRAS^G12D^. As recent data show [7], deciphering the biology of mutation‐specific pancreatic cancer is of utmost significance because particular KRAS mutations are associated with distinct clinical outcomes and malignant biological features. Importantly, Li et al. identify STAT5 as a novel potential therapeutic target paving the way towards developing combinatorial strategies for selective targeting of both STAT5 and KRAS^G12D^ in pancreatic cancer [8, 9] (Figure 1), hence offering hope in the hunt for a treatment that could wipe out the disease.

## Author Contributions

**Kostas A. Papavassiliou:** conceptualization (lead), data curation (lead), writing – original draft (lead). **Christos Adamopoulos:** conceptualization (equal), data curation (equal), writing – original draft (equal). **Athanasios G. Papavassiliou:** conceptualization (lead), data curation (lead), supervision (lead), writing – review and editing (lead).

## Conflicts of Interest

The authors declare no conflicts of interest.

## Data Availability

Data sharing not applicable—no new data generated.
